# Dual‐energy CT for proton therapy: Impact of advanced slice‐wise patient‐thickness estimation methods for improved stopping‐power prediction

**DOI:** 10.1002/acm2.70630

**Published:** 2026-05-23

**Authors:** Julian Schwengfelder, Nils Peters, Patrick Wohlfahrt, Christian Richter

**Affiliations:** ^1^ OncoRay – National Center for Radiation Research in Oncology Faculty of Medicine Dresden Saxony Germany; ^2^ Helmholtz‐Zentrum Dresden‐Rossendorf Institute of Radiooncology – OncoRay Dresden Saxony Germany; ^3^ Department of Radiation Oncology University of Washington & Fred Hutch Cancer Center Washington Seattle USA; ^4^ Siemens Healthineers Varian, Cancer Therapy Imaging Forchheim Bavaria Germany; ^5^ Department of Radiotherapy and Radiation Oncology Faculty of Medicine and University Hospital Carl Gustav Carus TUD Dresden University of Technology Dresden Saxony Germany; ^6^ German Cancer Consortium (DKTK) Partner Site Dresden and German Cancer Research Center (DKFZ) Heidelberg Germany

**Keywords:** dual‐energy computed tomography, proton therapy, stopping‐power ratio, thickness estimation

## Abstract

**Background:**

The direct prediction of stopping‐power ratio (SPR) from dual‐energy CT (DECT) has become gold‐standard in proton therapy. Remaining uncertainties due to patient‐size‐specific CT number variations are mitigated by calibration factors based on patient size defined as water‐equivalent thickness.

**Purpose:**

To improve SPR prediction, two slice‐wise thickness estimation methods (TEM‐B1 and ‐B2) were compared with the previously used one (TEM‐A).

**Methods:**

TEM‐A is using the maximum attenuation projections in *x*‐ and *y*‐direction, while TEM‐B1 and ‐B2 incorporate all voxels of the object to better describe non‐elliptical geometries. Simplified geometries were used to investigate TEM dependencies on several parameters (e.g., object shape, rotation). TEMs were then applied to DECT scans of cylindrical acrylic phantoms with varying diameters and to patient data. Clinical treatment plans were recalculated on generated TEM‐specific SPR datasets, and the impact of different estimated thicknesses on SPR was assessed.

**Results:**

In contrast to TEM‐A, TEM‐B1 and ‐B2 demonstrated robustness to object shape and rotation. Couch attenuation affected all evaluated TEMs with TEM‐A being most affected. For patient scans, TEM‐B1 and B2 agreed closely but differed from TEM‐A, especially in high diameters. In obese patients, this leads to relative proton range deviations up to 0.3% when comparing TEM‐B1 and TEM‐A. In the sensitivity analysis, TEM‐B1 and ‐B2 maintained SPR uncertainties below ±3% even for cortical bone.

**Conclusions:**

TEM‐B1 and ‐B2 reduced deviations in thickness estimation and increased robustness to object shape, overcoming the limitations of TEM‐A and improving SPR prediction accuracy.

## 1 INTRODUCTION

Accurate prediction of stopping‐power ratio (SPR) is essential of proton therapy, as it directly results in uncertainty in dose calculation. Conventionally, SPR is derived from single‐energy computed tomography (SECT) scans through a Hounsfield look‐up table (HLUT) conversion.^1^ Using dual‐energy computed tomography (DECT) with two distinct x‐ray spectra, tissue parameters such as relative electron density (RED) and effective atomic number (EAN) can be directly determined through a calibrated linear weighting of the two respective DECT datasets.^2^, ^3^ The RED, together with the mean excitation energy, obtained through a heuristic conversion from EAN,^4^, ^5^ serves as an essential input parameter for the Bethe equation used for voxel‐wise SPR calculation^3^, ^6^, ^7^, ^8^ – a primary advantage over HLUT methods.^9^, ^10^

Multiple studies demonstrated that DECT‐based direct SPR prediction substantially reduces the uncertainty in SPR compared to SECT‐based HLUT approaches.^11^, ^12^, ^13^, ^14^, ^15^, ^16^ However, one of the primary sources of uncertainty in SPR prediction remains the variability in CT numbers (CTNs) across different object sizes due to beam hardening conditions differing between patients.^17^, ^18^, ^19^ Beam hardening correction for bones is advised to partly mitigate this effect.^20^, ^21^, ^22^ Consideration of patient diameter in the calculation of RED and EAN has been shown to further reduce CTN variation,^7^ allowing for a personalized tissue characterization. The current thickness estimation within the scanner relies on assessing maximum attenuation projections in the *x*‐ and *y*‐directions, enabling a slice‐wise determination of the patient size, more precisely the water‐equivalent thickness (WET) for diagnostic x‐rays.^17^ This inherently oversimplifies the patient geometry, assuming an elliptical shape. Uncertainties in WET estimation directly introduce errors in the assigned calibration factor, subsequently propagating into SPR prediction deviations. With the high precision requirements of proton therapy, in combination with the endeavor to further reduce range uncertainties, addressing these limitations is a straightforward opportunity to further reduce uncertainties in SPR prediction and thus enable more accurate, patient‐specific dose calculation.

In this study, two advanced TEMs that integrate all pixels of the respective CT slice into the thickness estimation process were introduced to enhance the accuracy and reliability of SPR prediction for proton therapy. These new methods were compared with the existing TEM on different complexity levels, analyzed their influencing factors, and examined the impact of WET variability on SPR prediction. All TEMs were used to generate SPR datasets for retrospective patient data to evaluate the influence of different WET estimations on proton range computation and dose distribution under clinical conditions.

## 2 MATERIALS AND METHODS

### 2.1 Image acquisition

For this investigation, all CT scans were acquired with a dual‐spiral DECT scanner (SOMATOM Definition AS Open, Siemens Healthineers, Forchheim, Germany) using 80 and 140 kVp x‐ray tube voltage and were reconstructed with a slice thickness of 2 mm using an iterative reconstruction algorithm (SAFIRE, strength 3) with the quantitative kernel Qr40 containing beam hardening correction for bone.^23^ For the determination of the SPR, the commercially available DirectSPR algorithm (Siemens Healthineers) was used.

### 2.2 Thickness estimation methods

The CTN H in Hounsfield units (HU), represented as pixel values in the reconstructed image slices, is directly associated with μ as the material's linear x‐ray attenuation coefficient relative of water^9^:

(1) $$H=\left(\mu -1\right)\cdot 1000\;\mathrm{HU}$$

The slice‐wise estimation of the object's WET for diagnostic x‐rays relies on the low‐energy 80 kVp CT scan, following the method of TEM‐A (see Section 2.2.1). The overall object WET then serves as input parameter for deriving calibration $\alpha_{\mathrm{RED}}\left(\mathrm{WET}\right)$ and $\alpha_{\mathrm{EA}N^{3.1}}\left(\mathrm{WET}\right)$ (cf. Figure 3). As these factors are WET‐specific, they account for variations in the low‐ and high‐energy CTN due to WET‐specific beam hardening. The DECT datasets are then superimposed with the weighting factors $\alpha_{\mathrm{RED}}$ and $\alpha_{\mathrm{EA}N^{3.1}}$ to derive the voxel‐vise RED and EAN, respectively.^17^ Subsequently, the resulting RED and EAN are used to compute the SPR via the Bethe equation.^6^

#### 2.2.1 Existing thickness estimation method (TEM‐A)

TEM‐A, which is natively directly implemented in the projection domain, calculates the overall WET of a scanned object by analyzing its maximum attenuation in horizontal and vertical dimension.^17^ In the image domain, this can be described by identifying the maximum attenuation extent along both directions (i.e., rows and columns) by summing the products of the linear x‐ray attenuation coefficients relative to water and the corresponding pixel dimensions. This approach follows the concept of effective diameter estimation as defined in AAPM Report 204,^24^ with the adaption that geometric extents are weighted by linear attenuation coefficients to better reflect the attenuation properties of the scanned object. For each image slice, the overall object WET is determined as the geometric mean of these maximum extents:

(2) $$\mathrm{WE}T_{\mathrm{TEM}-A}=\sqrt{\max\left\{\sum_{i=0}^{m}\mu_{i,j}\cdot a_{i}\right\}\cdot \max\left\{\sum_{j=0}^{n}\mu_{i,j}\cdot a_{j}\right\}}$$

with a denoting x‐ and y‐dimension of each pixel (i,j) in x‐ and y‐direction, respectively.

This approach provides a straightforward WET estimation but has inherent limitations as it only incorporates two perpendicular pixel lines and thereby oversimplifies the object shape, assuming a degree of symmetry and homogeneity that represents only a rough estimate for real patients.

#### 2.2.2 Advanced thickness estimation methods (TEM‐B1 and ‐B2)

To address these limitations, two novel image‐based TEMs were developed—TEM‐B1 and TEM‐B2—that incorporate all geometric and density information in the respective axial CT slice into the thickness estimation process, allowing to assess the attenuating area of the object.

TEM‐B1 accumulates all attenuation values within the scan object, weighted according to their pixel size, resulting in an output representing an area. Subsequently, the diameter of a circle with an equivalent area is determined:

(3) $$\mathrm{WE}T_{\mathrm{TEM}-B1}=2\cdot \sqrt{\frac{\sum_{i=0}^{m}\sum_{j=0}^{n}\;\mu_{i,j}\cdot \left(a_{i}\cdot a_{j}\right)}{\pi }}$$

Of note, this image‐based approach is similar to a projection‐based approach, where the signal from each detector element, multiplied by the length of the detector element, gives the water‐equivalent path length in x‐ray direction. Multiplying this by the detector element's width yields the area integral (total attenuation per row). Summing all area integrals provides the total object's attenuation area, from which the diameter of the equivalent area circle can be calculated.

TEM‐B2 is mathematically similar to TEM‐B1 with a subtle difference: Here, the water‐equivalent area of each pixel is calculated as the product of its WET dimensions in the *x*‐ and *y*‐directions. These water‐equivalent dimensions are determined by multiplying the pixel's linear x‐ray attenuation coefficient (relative to water) with the pixel size in the respective direction. All pixel water‐equivalent areas are then summed up to determine the total water‐equivalent area of the object. Subsequently, the diameter of a circle with an equivalent area is computed:

(4) $$\mathrm{WE}T_{\mathrm{TEM}-B2}=2\cdot \sqrt{\frac{\sum_{i=0}^{m}\sum_{j=0}^{n}\;\left(\mu_{i,j}\cdot a_{i}\right)\cdot \left(\mu_{i,j}\cdot a_{j}\right)}{\pi }}$$

In comparison to TEM‐B1, this increases sensitivity to variations in tissue composition, as TEM‐B2 incorporates each pixel's attenuation coefficient quadratically, making it particularly responsive to structures with attenuation properties differing from soft tissue. However, TEM‐B2 is limited to the image domain, as it requires a reconstructed CT image as input, whereas TEM‐B1 could also be implemented in the projection domain.

### 2.3 TEM assessment on different complexity levels

**FIGURE 1:**
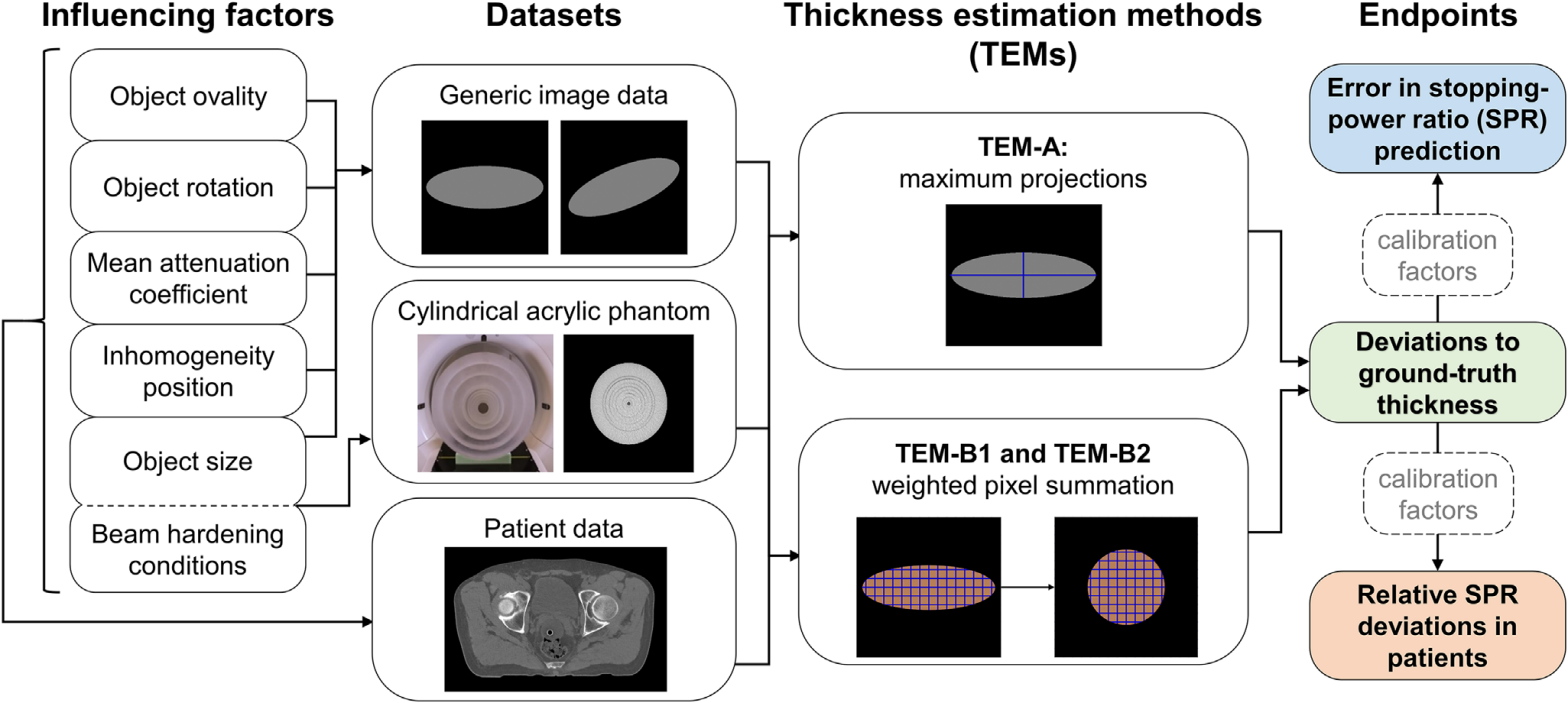
Flowchart illustrating the systematic investigations used to evaluate the thickness estimation methods (TEM) dependencies and their influence on stopping‐power ratio (SPR) prediction.

All TEMs were comprehensively tested on datasets of increasing complexity—generic image data, phantom scans, and patient data—to systematically investigate their dependencies on various parameters (Figure 1).

#### 2.3.1 Generic image data

First, all TEMs were implemented and tested on generic image data to analyze the TEMs under idealized conditions, without the presence of beam hardening effects. Simplified geometries, that is, circles and ellipses, were created using python scripting, with pixel values representing the attenuation coefficient μ (relative to water). The ground‐truth diameter was defined as the geometric mean of the ellipse's major and minor axes, following Cook et al.’s definition of the effective ellipse diameter,^25^ multiplied by the object's overall mean attenuation coefficient. In a first step, the geometries were uniformly assigned with the linear attenuation coefficient of water to assess the influence of object size, shape, and rotation. In a second step, varying attenuation coefficients (ranging from 0.9 to 1.1 relative to water) were used and in addition, inhomogeneities with different positions (centered and off‐center) were introduced.

#### 2.3.2 Cylindrical acrylic phantom

To better reflect clinical scenarios and account for beam hardening effects, the TEMs were applied to DECT scans of cylindrical acrylic phantoms with diameters ranging from 75 to 400 mm. The estimated WETs were compared to the ground‐truth WETs of the phantoms, calculated by multiplying the phantom's geometric diameter with the linear x‐ray attenuation of the phantom material (polymethyl methacrylate).

Due to the introduced changes in the WET estimation process, new RED and EAN calibrations for TEM‐B1 and ‐B2 were required. Therefore, DECT scans of the same phantom, including tissue‐equivalent materials from Gammex (Sun Nuclear Corporation, Middleton, WI, USA) and CIRS (Sun Nuclear Corporation, Norfolk, VA, USA), were acquired. CTNs from high‐ and low‐energy DECT scans were extracted within defined ROIs inside the tissue‐equivalent surrogates. The scans of surrogates with high CTN differences, especially bone‐equivalent structures, were used to generate TEM‐specific calibration curves for RED and EAN, following Möhler et al.^9^

#### 2.3.3 Patient data

For the most complex yet clinical scenarios, the TEMs were retrospectively applied to representative patient scans, including head and neck (H&N), prostate, and brain cancer, with three cases from each entity (approved by the local ethics committee). The patient cohort was intentionally selected to cover a wide spectrum of clinical scenarios, including a range of patient diameters and cases with known sources of beam hardening artifacts, such as dental materials. The slice‐wise estimated WETs for all TEMs were compared, along with the respective calibration factors for RED and EAN determination. Subsequently, TEM‐specific SPR datasets were generated, and the impact of different TEMs was analyzed through voxel‐wise SPR comparisons.

Additionally, clinical treatment plans were recalculated on all SPR datasets using the treatment planning system RayStation 2023B (RaySearch Laboratories, Stockholm, Sweden) utilizing the implemented Monte Carlo dose calculation algorithm with the direct import tool for SPR maps, which are then translated into base materials within the TPS. Details on the evaluated clinical treatment plans are provided in the Table S1. For each treatment field, spot‐wise proton range differences in the high‐dose areas were computed from integrated depth dose curves, indicated by the shift of the 80% of the absorbed dose distal the clinical target volume, using an in‐house python routine. To further contextualize the findings in terms of clinical relevance, dose differences are reported as an additional, complementary metric.

### 2.4 Sensitivity analysis to mitigate TEM deviations to SPR level

In a sensitivity analysis, similar to the approach presented in Peters et al.,^17^ the impact of WET variability on the resulting SPR was assessed. For this purpose, DECT scans of the same cylindrical acrylic phantom as used in 2.3.2 with varying diameters and different tissue‐equivalent surrogates – and thus varying WETs – were used. To ensure that the observed SPR deviations were solely due to differences in the thickness estimation process, the images were cropped to exclude the patient couch. It should be noted that couch removal by image cropping was applied exclusively to enable an unconfounded assessment of intrinsic TEM performance.

In the first step, RED and EAN calibration factors were derived from the TEMs calibration curves for the respective WETs. These calibration factors, along with the low‐ and high‐energy‐CTN of various tissue‐equivalent surrogates (lung inhale and exhale, muscle, brain, trabecular, and cortical bone; Gammex [Sun Nuclear Corporation, Middleton, WI, USA]) were used to calculate the corresponding nominal SPR.

In the second step, WET was modified by the maximum deviations in WET estimation observed between different TEMs in scans of the cylindrical phantom (see Section 2.3.2). The WET modifications included both increases and decreases corresponding to the maximum observed deviation. SPR was then recalculated using calibration factors adjusted for a modified WET, while keep ing the CTNs constant.

## 3 RESULTS

### 3.1 Generic image data

For circular shapes and horizontally aligned ellipses, all TEMs accurately estimated the known ground‐truth. For rotated ellipses, TEM‐A showed deviations, while TEM‐B1 and ‐B2 remained accurate (cf. Figure S1), demonstrating their robustness to rotation and object geometry. TEM‐A also showed substantial errors when inhomogeneities were added off‐centered to the generic shapes. Variations in the objects mean attenuation coefficients led to variation in the WET estimations between the different approaches, highlighting the need for TEM‐specific calibration curves due to their individual estimation processes.

### 3.2 Image data of cylindrical acrylic phantom

**FIGURE 2:**
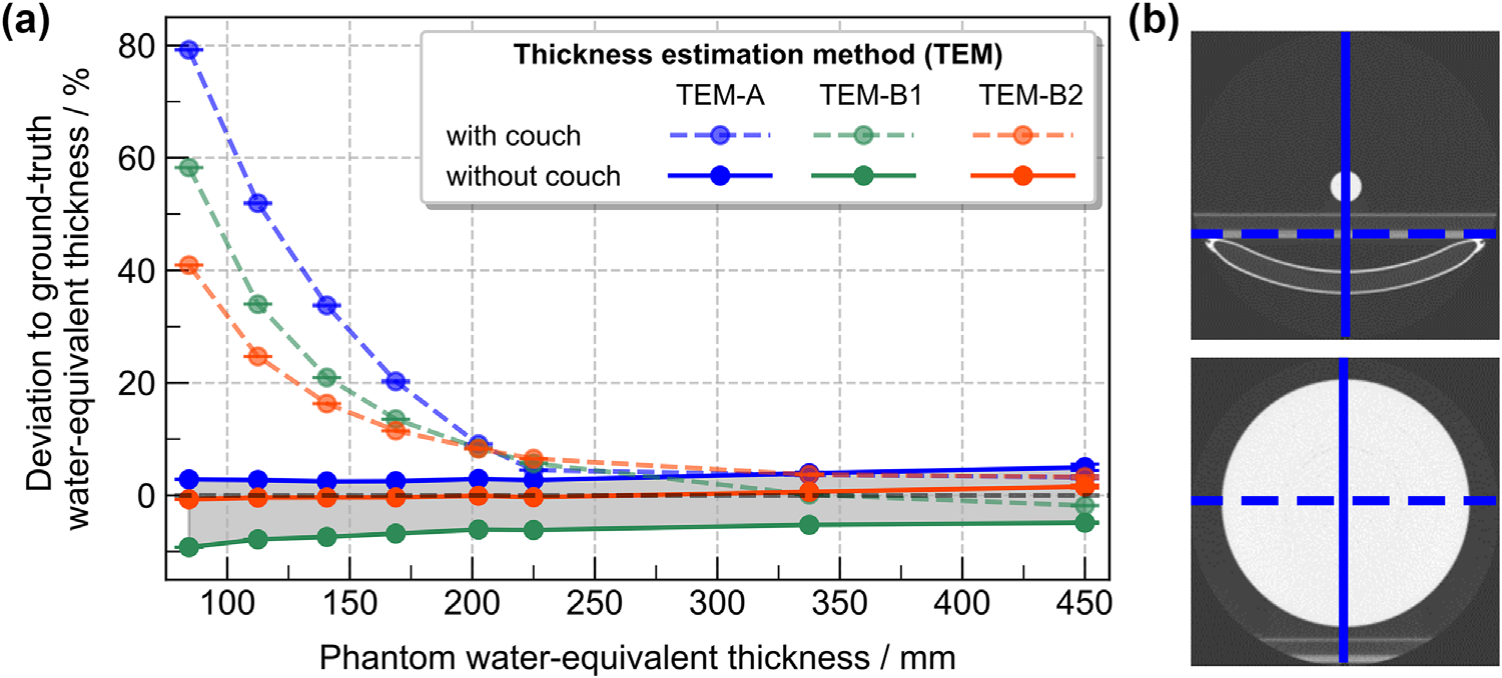
(a) Deviations between the estimated water‐equivalent thickness (WET) and ground‐truth phantom WET for the thickness estimation methods (TEMs), with and without the influence of the patient couch. The gray‐shaded area highlights the maximum deviation between TEMs. (b) Visualization of TEM‐A's determined maximum projections in *x* (dashed) and *y* direction (solid blue line) for two example phantom diameters.

For small phantom diameters of the cylindrical acrylic phantom (WET < 200 mm), the WET was overestimated compared to the ground‐truth for all TEM approaches due to additional couch attenuation, with TEM‐A being most affected (Figure 2a). This effect is more pronounced for smaller scan objects, where the couch attenuation represents a larger proportion of the overall attenuation. TEM‐A's reliance on maximum projection profiles in *x* and *y* directions amplified this error for small objects where the maximum *x* projections aligned with the couch surface, thus being shifted out of the phantom itself (Figure 2b). TEM‐B2 performed slightly better than TEM‐B1.

To evaluate the intrinsic TEMs performance without confounding effect of couch attenuation, the couch was removed from the image content. After this refinement, WET deviations among the TEMs were within 10%, with TEM‐B2 showing the closest agreement to the ground‐truth.

**FIGURE 3:**
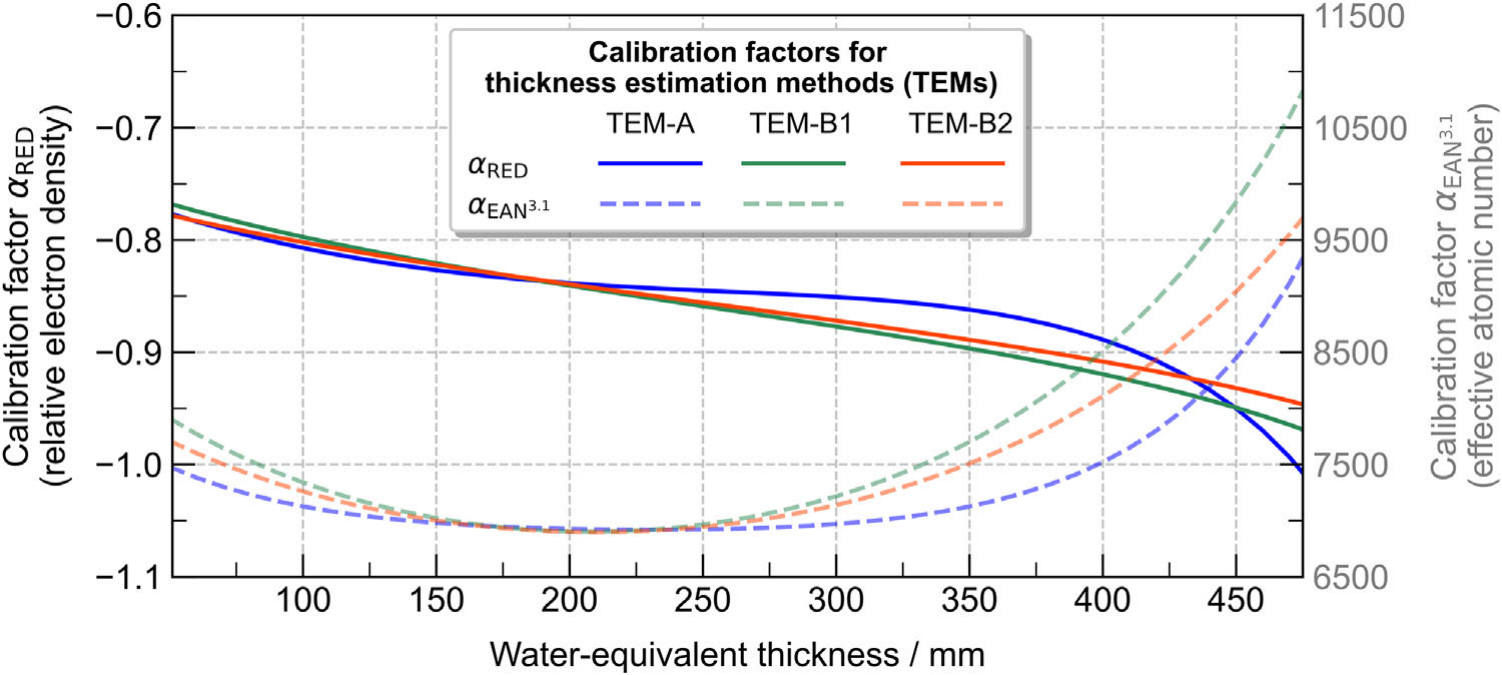
Size‐dependent calibration factors for relative electron density (RED) $\alpha_{\mathrm{RED}}$ and effective atomic number (EAN) $\alpha_{\mathrm{EA}N^{3.1}}$ across the thickness estimation methods (TEMs).

The determined RED and EAN calibration curves for TEM‐B1 and B2 were less steep compared to TEM‐A, especially for larger phantom geometries (Figure 3).

### 3.3 Patient data analysis

Applying the investigated TEMs to patient scans resulted in different WET estimations for the respective approaches. TEM‐B1 and TEM‐B2 showed similar WET estimations, whereas TEM‐A notably differed, particularly for larger patient diameters (> 300 mm). On the level of RED and EAN, these differences were partially mitigated by the TEM‐specific calibration factors. For moderate WET values (< 300 mm), RED and EAN calibration factors were comparable across all TEMs as shown for three exemplary patients (cf. Figure S2). However, particularly in obese patients and at high WETs (> 300 mm), calibration factors of TEM‐A substantially differed. Compared to TEM‐B1 and ‐B2, TEM‐A was notably more affected by beam hardening artifacts, such as those from dental materials, resulting in inflated WET estimations. However, their influence on the slice‐specific calibration factors remained negligible across all affected cases.

**FIGURE 4:**
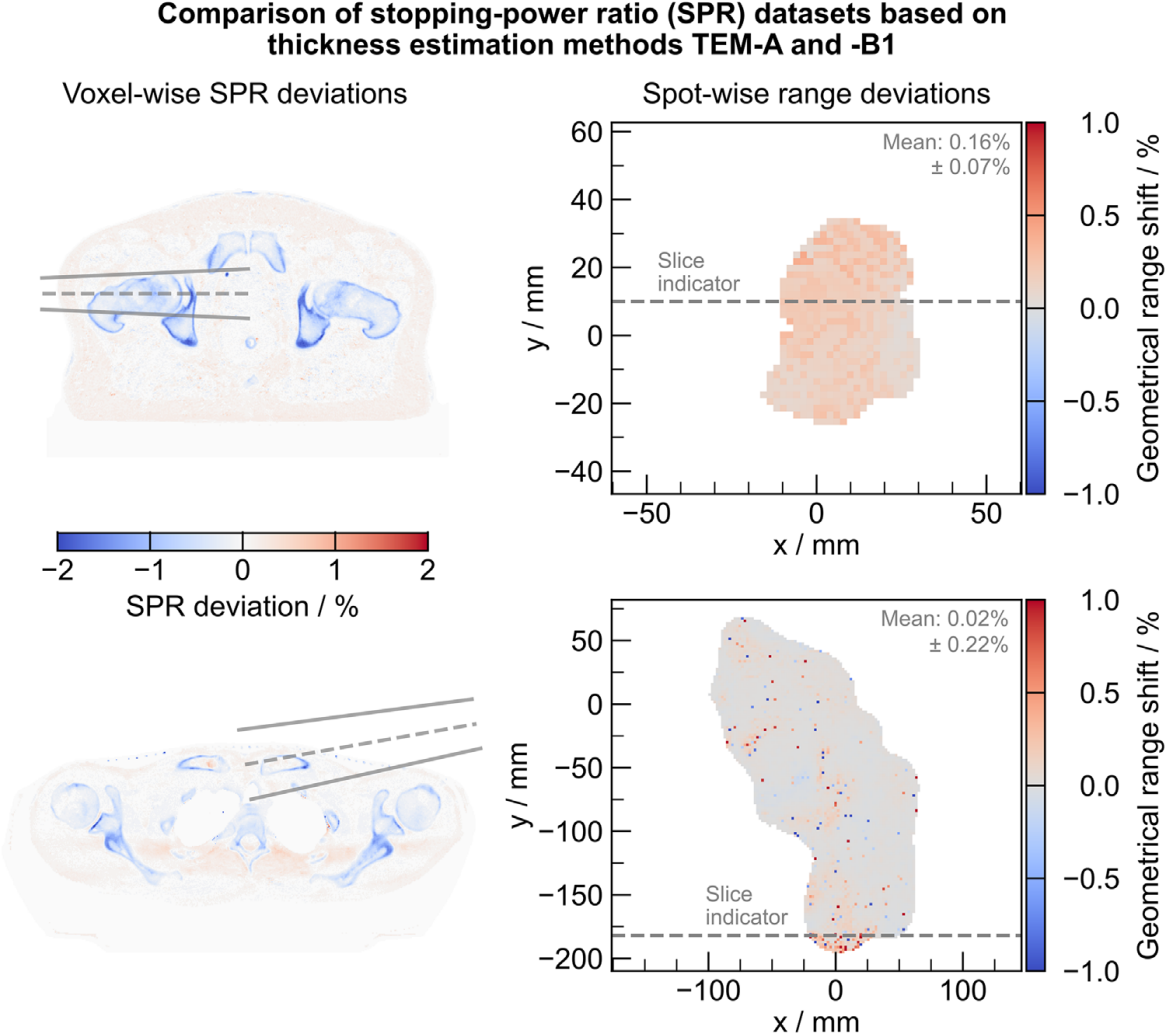
Comparison of stopping‐power ratio (SPR) datasets based on TEM‐A and TEM‐B1 for an exemplary prostate cancer (top row) and head‐and‐neck cancer patient (bottom row). (Left) Voxel‐wise SPR deviations for a representative axial CT slice. (Right) Spot‐wise geometric range deviations in the beam's‐eye‐view direction.

As the primary endpoint of this study, voxel‐wise SPR differences were evaluated first. Owing to similar calibration factors, SPR datasets based on TEM‐B1 and B2 were nearly identical, with differences to TEM‐A most pronounced in bony structures. In the voxel‐wise SPR comparison, the absolute SPR deviations between TEM‐B1 and TEM‐A reached up to 2% in cortical bone for an obese patient (Figure 4), whereas the comparison of TEM‐B1 and ‐B2 showed negligible differences (Figure S3).

**FIGURE 5:**
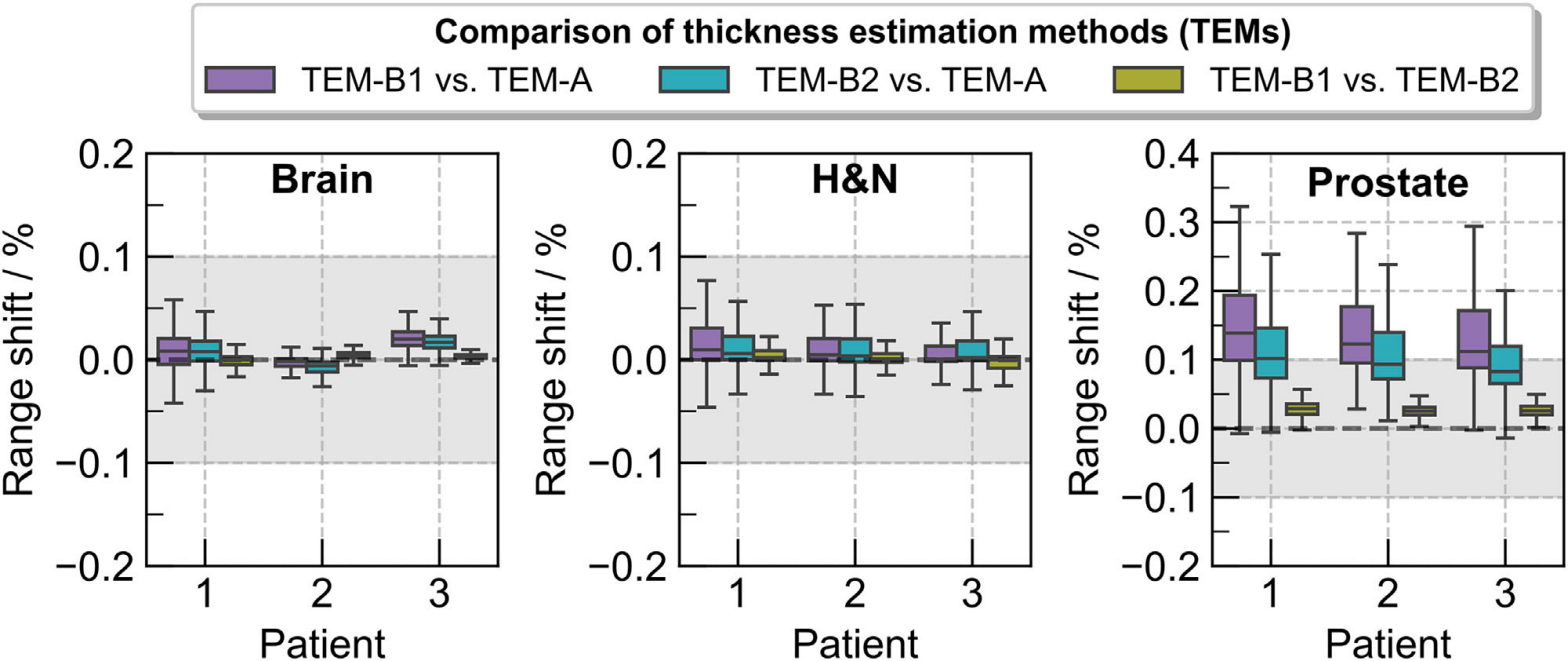
Boxplots showing patient‐specific relative proton range differences (combined for all treatment fields) when comparing the same treatment plan calculated on stopping‐power ratio (SPR) datasets derived from the three thickness estimation methods (TEMs). The gray‐shaded region represents the range of ±0.1%.

These SPR differences directly translated into proton range shifts: Across all patients the spot‐wise range comparison between TEM‐B1 and ‐B2 showed neglectable small range shifts, confirming the strong agreement between these two TEMs. In contrast, consistently larger deviations were evident between TEM‐B1 and TEM‐A (Figure 4). In brain and H&N cohorts, relative range shifts between TEM‐B1 and TEM‐A remained within ±0.1%, while prostate cases (larger patient diameter) showed deviations up to ±0.3%, with higher proton ranges for TEM‐B1 (Figure 5). For an obese prostate cancer patient, a mean water‐equivalent range shift of 0.32 mm was found when comparing TEM‐B1 and TEM‐A. This translated into absolute dose differences up to 2% relative to the prescribed dose in the dose fall‐off region distal to the target volume (Figure S4). In contrast, dose differences between TEM‐B1 and ‐B2 were < 0.5%. For brain and H&N cohorts, dose differences for TEM‐B1 versus TEM‐A were constantly below 0.3%.

### 3.4 Sensitivity analysis

**FIGURE 6:**
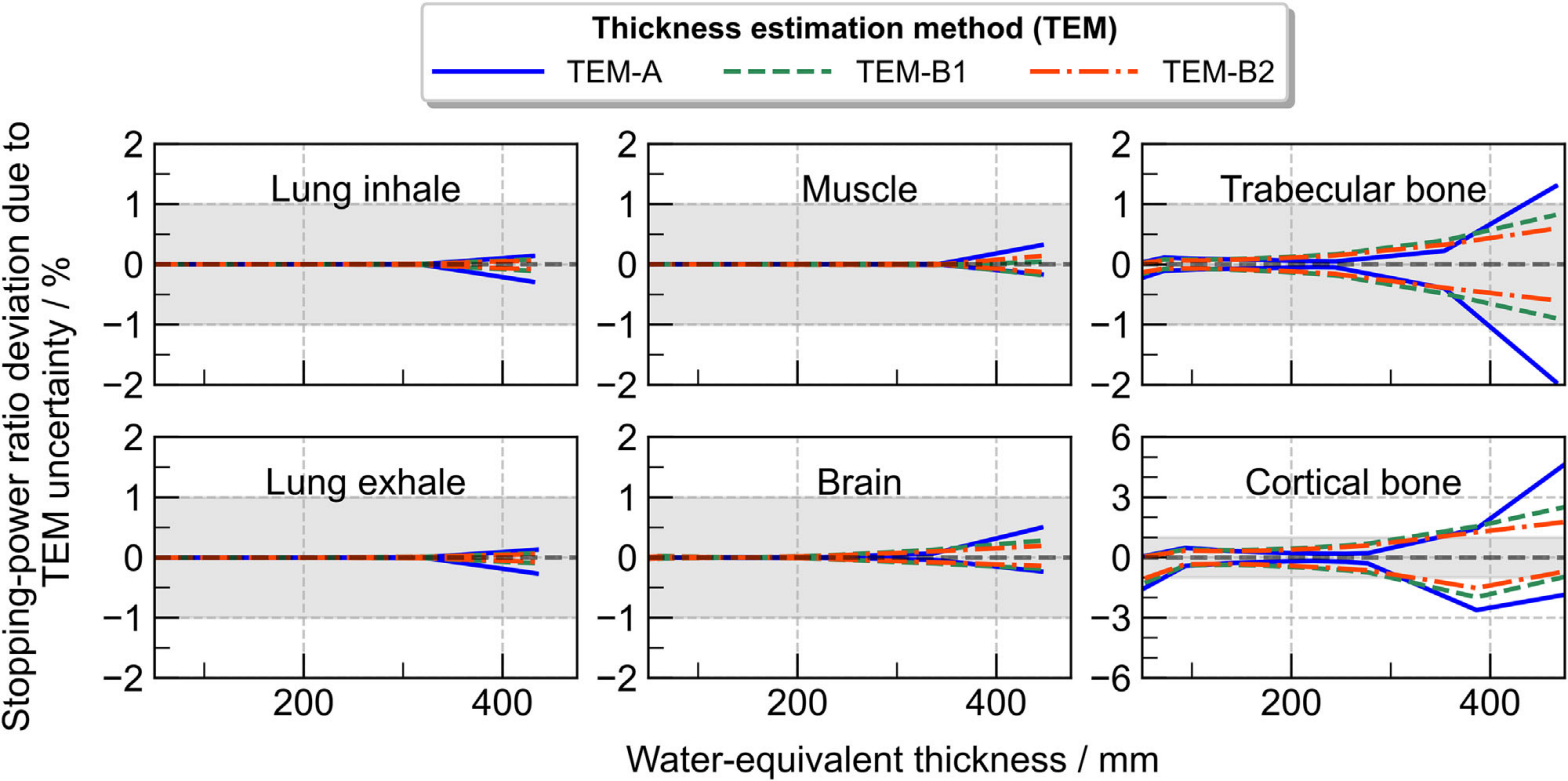
Stopping‐power ratio deviations from water‐equivalent thickness estimation uncertainty, based on the maximum thickness estimation method (TEM) deviations observed in the cylindrical acrylic phantom scans. The gray‐shaded region represents the range of ±1%.

SPR predictions for lung and soft tissues were relatively robust to WET variability across all three TEMs (Figure 6). At higher WETs, SPR uncertainty increased due to TEM variability but remained below ±1% for these tissues across all TEMs, with TEM‐A exhibiting the highest uncertainty. In contrast, denser materials like trabecular and cortical bone were more affected by WET variability. For cortical bone, SPR deviations due to WET uncertainty exceed ±3% for large WETs (> 400 mm) with TEM‐A, whereas TEM‐B1 and TEM‐B2 stayed within 3%, demonstrating greater robustness to WET variations.

## 4 DISCUSSION

This study introduces two image‐based TEMs (TEM‐B1 and TEM‐B2) for a more robust and accurate DECT‐based direct SPR prediction using size‐specific calibration factors. By incorporating all anatomical information from the patient in each image slice, the proposed methods directly address the limitations of the existing approach TEM‐A, which is more susceptible to irregular shapes, imaging artifacts and object rotation. The proposed methods showed better robustness, resulting in reduced SPR prediction errors, especially under challenging conditions. In patient data, deviations in WET estimation were more pronounced compared to phantom studies, driven by greater complexity, such as material inhomogeneities, irregular shapes and intricate beam hardening scenarios. Beam hardening is particularly pronounced in bony structures, where strong absorption of low‐energy photons is amplified, increasing CTN variability between high‐ and low‐energy DECT. Importantly, with the size‐dependent determination of calibration factors, CTN variability due to beam hardening is mitigated. Hence, accurate determination of calibration factors is particularly important in bony regions, as underlined by the sensitivity analysis.

Previous studies showed that RED prediction uncertainty significantly affects SPR, making especially the RED calibration curve critical for accurate SPR predictions, while EAN uncertainty has a smaller impact.^5^, ^26^ TEM‐B1 and TEM‐B2 showed flatter RED calibration curves than TEM‐A, particularly for high WETs (> 400 mm). This translates to a more stable SPR prediction, especially for greater WETs, highlighting that the main benefit of the improved TEM arises in higher WET. However, it is important to mention that in clinical routine, for most patients, WETs are below 400 mm, with only severely obese patients having a larger thickness.

In our study, only the thickness estimation process and size‐specific calibration were modified for SPR prediction, limiting the number of factors contributing to SPR variations. For bone, Peters et al.^17^ reported a 1σ SPR uncertainty of 1.41% from size‐specific calibration and up to 0.24% from thickness estimation, resulting in a combined 1σ uncertainty of 1.43% based on uncertainty propagation. This represents one of the largest individual contributing factors to the overall range uncertainty, estimated at approximately 1.7%–2% for DECT‐based direct SPR prediction,^17^ compared to 3%–3.5% for conventional SECT‐HLUT approaches.^27^ In the voxel‐wise SPR comparison of our study, a 1σ SPR variation of approximately 0.7% was seen in bony structures. Assuming this as the only modified contributing factor, this corresponds to an overall range uncertainty of approximately 1.6%–1.8%. While modest in absolute terms, a reduction of up to 0.2 percentage points compared to the currently adopted clinical range uncertainties is meaningful given the high precision requirements of proton therapy and the ongoing effort to progressively reduce range uncertainties.

The algorithmic difference between TEM‐B1 and TEM‐B2 is solely based on the quadratic incorporation of attenuation coefficients in TEM‐B2. For the investigated patient scenarios, the differences between TEM‐B1's and ‐B2's estimated WETs remained negligible, as the overwhelming majority of pixels within the patient belong to soft tissue, where the linear attenuation coefficient relative to water is close to 1. In contrast to that, in body regions with a larger amount of lung, dense bone or even metal‐induced artifacts, WET estimations diverged, but were reconciled through dedicated RED and EAN calibration curves for TEM‐B1 and ‐B2 (Figure S2). This emphasizes the importance of the combination of precise WET estimation with accurate calibration. Notably, TEM‐A was more susceptible to such artifacts, as locally inflated attenuation values directly affect its maximum‐based WET estimation. However, as these deviations remain small relative to the overall patient WET, their influence on the calibration factors and consequently on the SPR was negligible in the present cohort.

To directly assess the clinical impact on range prediction for proton therapy, the presented methods were applied to the DECT‐based DirectSPR algorithm. While the dosimetric analysis thus is limited to the applied algorithm and the specific patient scenarios investigated, the findings regarding stability of the thickness estimation are generally applicable to all CT‐based tissue characterization methods considering patient thickness.

In the presented implementation, both TEM‐B1 and TEM‐B2 operate on reconstructed CT images and have a similar algorithmic complexity, resulting in no observable difference in computational time. A limitation of this image‐based TEM implementations is that WET is estimated slice‐wise, which neglects cross‐slice effects such as scatter or partial‐volume averaging. These effects can be captured in a projection‐based implementation, where all projections contributing to an axial image slice are accounted for size estimation. The presented method TEM‐B1 is generally transferable to the projection domain, where the total object's attenuation can be obtained directly from detector signals, whereas the TEM‐B2 approach always requires a reconstructed CT image. In addition to the consideration to cross‐slice effects, thickness estimation in the projection domain further increases computational efficiency. The scope of the present investigation was, however, to compare the TEM‐performance with respect to SPR prediction accuracy rather than computational efficiency.

## 5 CONCLUSION

Independent of the calibration, TEM‐B1 and TEM‐B2 demonstrated their ability to address the inherent limitations of TEM‐A, such as its sensitivity to shape irregularities and imaging artifacts. Both presented TEMs exhibited reduced SPR prediction uncertainties, especially in high‐WET scenarios and bony structures, leading to improved accuracy in proton range predictions. Given their improved robustness and accuracy, one of the TEM‐B should be considered for future implementation.

## AUTHOR CONTRIBUTIONS

**Julian Schwengfelder**: Conceptualization, methodology, software, validation, formal analysis, investigation, data curation, writing – original draft, writing – review and editing, visualization. **Nils Peters**: Resources, data curation, writing – review and editing. **Patrick Wohlfahrt**: Resources, data curation, writing – review and editing. **Christian Richter**: Conceptualization, methodology, validation, writing – review and editing, supervision, funding acquisition, supervision, project administration.

## FUNDING INFORMATION

For the present study, the authors received no financial support involved in the study design or materials used, nor in the collection, analysis and interpretation of data nor in the writing of the publication.

## ETHICS STATEMENT

The retrospective analysis of clinical patient data was performed under the ethical approval granted by the local ethics committee of OncoRay (BO‐EK‐248062024).

## CONFLICT OF INTEREST STATEMENT

OncoRay has an institutional research agreement with Siemens Healthineers. Patrick Wohlfahrt is an employee of Siemens Healthineers within research and development. The authors have no conflicts of interest to disclose.

## Supporting information



SUPPORTING INFORMATION

## Data Availability

There is no usable data to share.
