# Supplementary material for: Dual‐energy CT for proton therapy: Impact of advanced slice‐wise patient‐thickness estimation methods for improved stopping‐power prediction
Source: J Appl Clin Med Phys. 2026 May 23;27(5):e70630. doi: 10.1002/acm2.70630 (PMC13240181; doi:10.1002/acm2.70630)
Supplement: Supplementary file 1 — SUPPORTING INFORMATION [file ACM2-27-e70630-s001.pdf]

Supplement material - Dual-energy CT for proton therapy: Impact of advanced slice-wise patient-thickness estimation methods for improved stopping-power prediction

Supplement A: Results of thickness estimation methods assessment on generic image data

The investigated thickness estimation methods (TEMs) were systematically tested on generic image data where the ground-truth water-equivalent thickness (WET) was defined as the geometric mean of the ellipse's major and minor axes, multiplied by the object's overall mean attenuation coefficient.

The application of the three TEMs to generic image data revealed, that all TEMs are able to accurately estimate the ellipses effective WET, as long as the ellipse is aligned with the x- and y-axis of the images coordinate system. For rotated ellipses within the image, TEM-A showed deviations to the ground-truth WET, as TEM-A relies on the determination of the maximum projection in x- and y- direction (see Supplement Figure S1).

When varying the mean pixel values of a circular object, TEM-A was able to accurately reproduce the ground-truth WET. In contrast, differences emerged between TEM-B1 and TEM-B2 due to their distinct weighting principles. In this simplified case, TEM-B2 aligned with the ground-truth definition, while TEM-B1 appeared slightly less accurate. However, this discrepancy does not stem from a limitation of the TEM-B1 algorithm itself, but rather from the way the ground-truth WET was defined, which is mathematically equivalent to TEM-B2 for such geometries.

For objects containing inhomogeneities, TEM-A's WET estimation strongly depended on the position of the inhomogeneity. In comparison, TEM-B1 and TEM-B2 produced stable results across different inhomogeneity positions, though both showed deviations from the defined ground-truth. Overall, these findings emphasize the importance of TEM-specific calibration curves to properly account for the algorithmic differences.

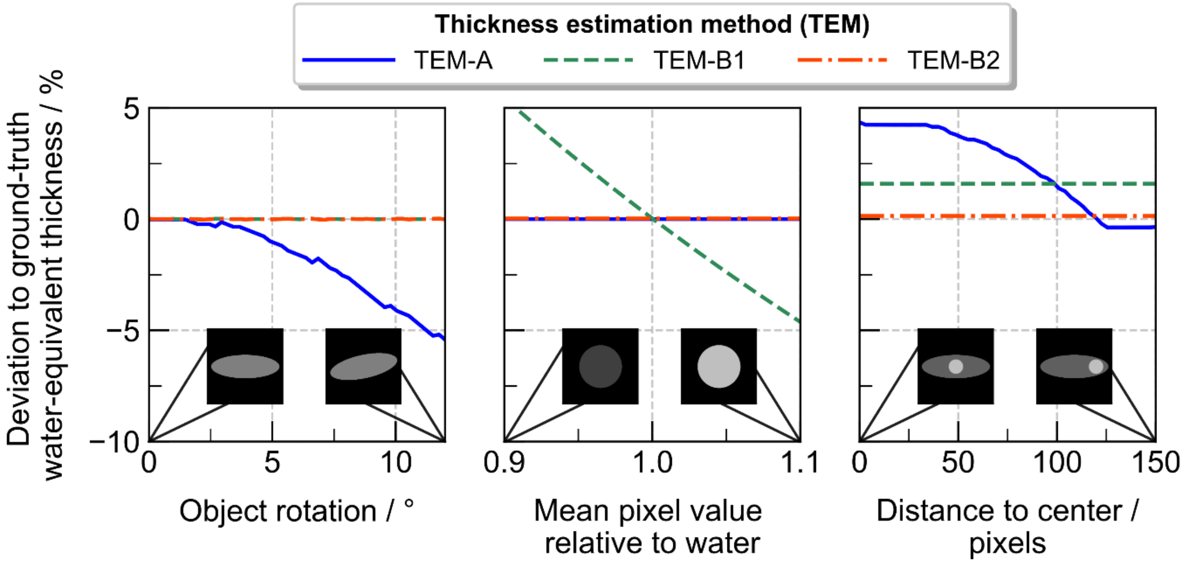

Supplement Figure S1: Deviations between the estimated water-equivalent thickness and ground-truth for generically generated objects for the three thickness estimation methods (TEMs). Results are shown for ellipse rotations (left), varying mean pixel values (center), and added inhomogeneities at different positions (right).

Supplement B: Results of thickness estimation methods assessment on patient data

The three TEMs were applied to retrospective patient data from brain, head and neck, and prostate cancer cases. TEM-B1 and TEM-B2 showed similar WET estimations, whereas TEM-A exhibited notable differences, particularly in regions with dental artifacts or larger patient diameters. Due to its algorithmic limitations, TEM-A produced substantially higher WET estimates in areas with very small patient diameters, such as the upper cranium. However, for intermediate patient WETs (<400 mm), these differences were mitigated by applying the respective calibration curves, resulting in similar calibration factors for relative electron density (RED) and effective atomic number (EAN).

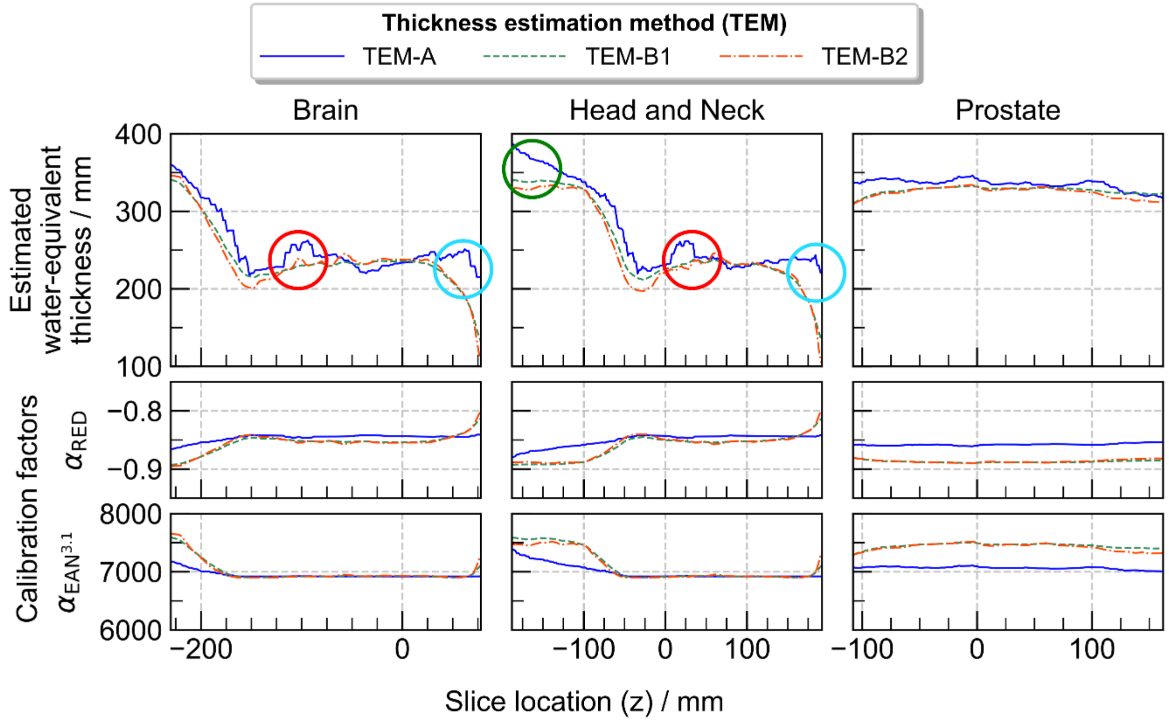

Supplement Figure S2: Upper row: Estimated water-equivalent thickness (WET) for three exemplary patient cases with lesions in the brain (left), head and neck region (center), and prostate (right). Red circles highlight regions of dental artifacts, blue circles the region of the patient's cranium and the green circle marks the area of the shoulders and upper lungs. Center and lower rows: Slice-wise calibration factors for relative electron density α_{RED} and effective atomic number $\alpha_{EAN^{3.1}}$ for the three thickness estimation methods (TEMs).

TEM-specific SPR datasets were generated and approved clinical treatment plans were recalculated on all SPR datasets using the treatment planning system RayStation 2023B (RaySearch Laboratories, Stockholm, Sweden) utilizing the implemented Monte Carlo dose calculation algorithm. The intensity-modulated proton therapy plans were based on pencil beam scanning and generated using robust optimization. Details on dose prescriptions and field configurations for the individual patient plans are provided in Supplement Table S1.

Supplement Table S1: Dose perceptions and field configurations for recalculated clinical treatment plans.

Patient		Dose prescription	Field configurations		
			Nr.	Gantry angle	Couch angle
Head	1	54 Gy in 30 fx	1<br>2<br>3	270°<br>90°<br>290°	10°<br>347°<br>85°
	2	30 Gy in 15 fx	1<br>2	295°<br>40°	15°<br>310°
	3	50 Gy / 60 Gy in 30 fx<br>(Simultaneous Integrated Boost (SIB))	1<br>2<br>3	250°<br>150°<br>300°	70°<br>350°<br>10°
Head and Neck	1	54.12 Gy / 69.96 Gy in 33 fx (SIB)	1<br>2<br>3	180°<br>45°<br>315°	0°<br>355°<br>5°
	2	50 Gy in 25 fx + 16 Gy in 8 fx<br>(sequential)	1<br>2<br>3	3°<br>80°<br>180°	0°<br>340°<br>0°
	3	54.12 Gy / 66 Gy in 33 fx (SIB)	1<br>2<br>3	0°<br>80°<br>179°	0°<br>345°<br>0°
Prostate	1	48 Gy / 60 Gy in 20 fx (SIB)	1<br>2	90°<br>270°	0°<br>0°
	2	48 Gy / 60 Gy in 20 fx (SIB)	1<br>2	90°<br>270°	0°<br>0°
	3	48 Gy / 60 Gy in 20 fx (SIB)	1<br>2	90°<br>270°	0°<br>0°

The voxel-wise stopping-power ratio (SPR) comparison revealed differences in SPR prediction when TEM-A was compared with TEM-B1 or TEM-B2. In bony structures, SPR differences of up to 2% were observed, resulting in proton range shifts (shown in chapter 3.3). In contrast, as shown in Supplement Figure S3, SPR differences between TEM-B1 and TEM-B2 were much smaller, leading to negligible range shifts. This highlights the strong comparability of TEM-B1 and TEM-B2.

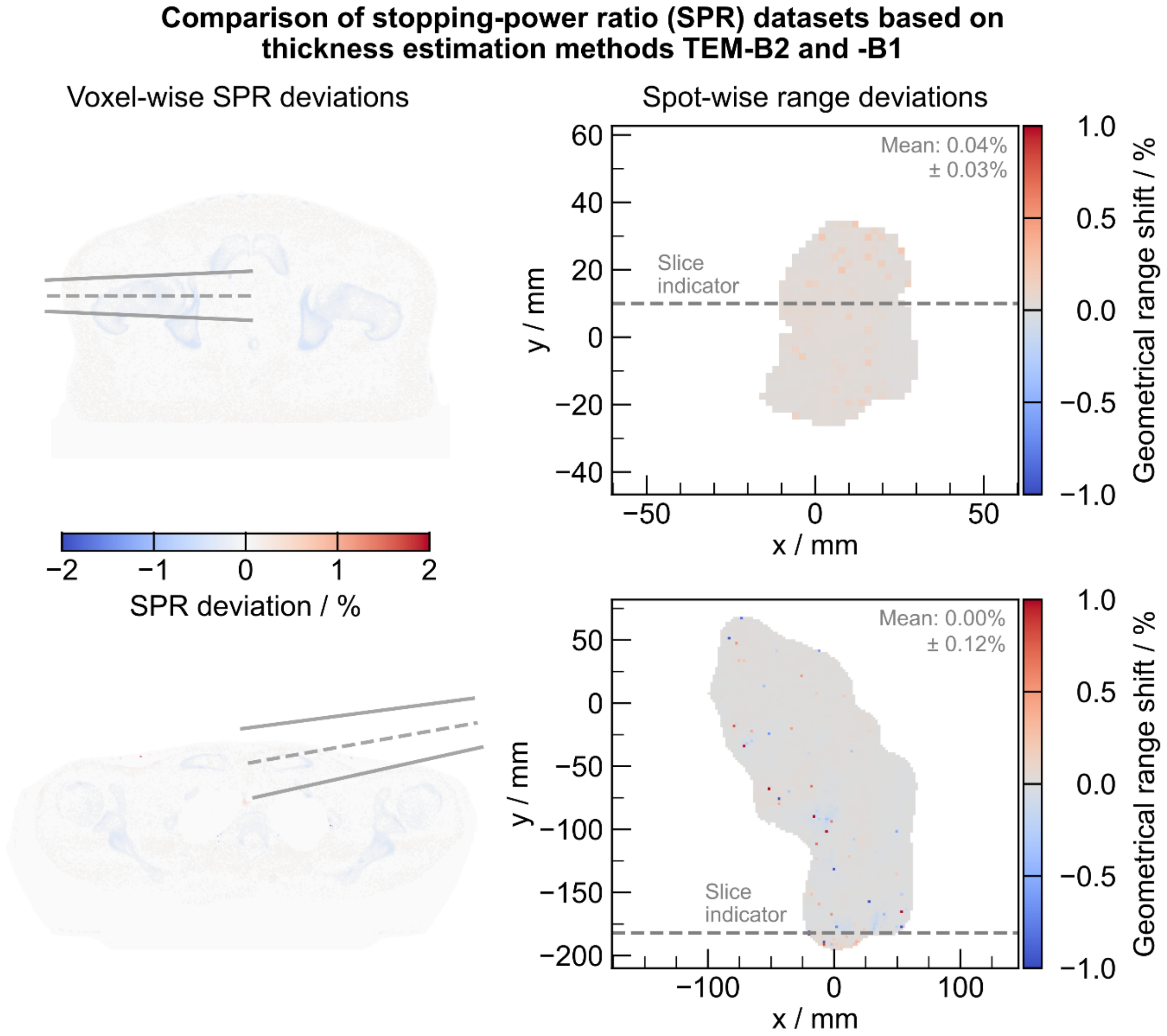

Supplement Figure S3: Comparison of stopping-power ratio (SPR) datasets based on TEM-B1 and TEM-B2 for an exemplary prostate cancer patient (top row) and head-and-neck cancer patient (bottom row). (Left) Voxel-wise SPR deviations for a representative axial CT slice. (Right) Spot-wise geometric range deviations in the Beam's-Eye-View direction.

The differences in SPR prediction among the TEMs result in proton range variations, which directly translate into dose differences when clinical treatment plans are recalculated on the different SPR datasets. Dose differences relative to the prescribed clinical dose reached up to 2% when comparing TEM-B1 and TEM-A, as demonstrated for an obese prostate cancer patient with a 60 Gy dose prescription. In contrast, comparisons between TEM-B1 and TEM-B2 revealed only minor dose differences.

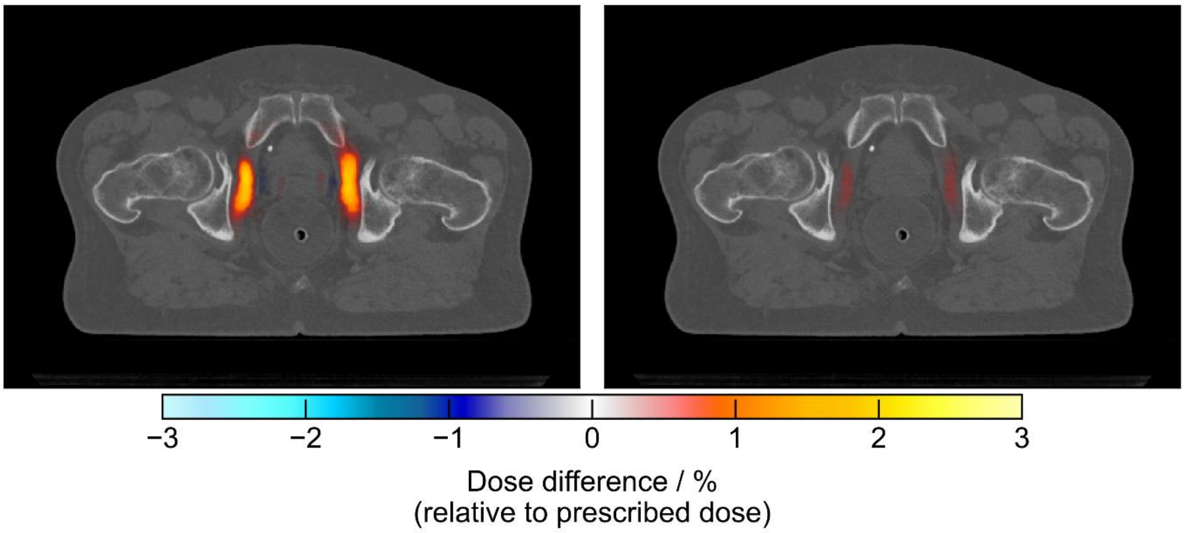

Supplement Figure S4: Dose differences relative to prescribed dose when comparing a) thickness estimation method (TEM)-B1 and TEM-A as well as b) TEM-B1 and -B2 for an exemplary obese prostate cancer patient (Patient 1 from the prostate cohort, dose prescription of 60 Gy).
